# GSH-Responsive Nano-Photosensitizer for Potentiating Photodynamic Therapy Through Multi-Pronged Synergistic Upregulation of Ferroptosis Sensitivity

**DOI:** 10.3390/antiox14040407

**Published:** 2025-03-28

**Authors:** Yunong Ma, Kexin Xu, Jing Feng, Xi Zhao, Peilin Tian, Jiayang Luo, Luyao Xu, Jiaxing Song, Cuixia Lu

**Affiliations:** 1 Guangxi Key Laboratory of Special Biomedicine, School of Medicine, Guangxi University, Nanning 530004, China; mynnong@163.com (Y.M.); echooo@st.gxu.edu.cn (K.X.); fengj@st.gxu.edu.cn (J.F.); zxnl2022@163.com (X.Z.); tianpl1216@st.gxu.edu.cn (P.T.); jiayungl@st.gxu.edu.cn (J.L.); luluna@st.gxu.edu.cn (L.X.); 2 Cell and Immunology Laboratory, Medical Research Centre, School of Life Sciences and Medical Engineering, Guangxi Medical University, Nanning 530021, China

**Keywords:** ferroptosis, chlorquinaldol, photodynamic therapy, GPX4, lipid peroxidation, oxidative stress

## Abstract

Impeded by the limited light penetration of photodynamic therapy (PDT) to tissues and the hypoxic environment of solid tumors, the clinical therapeutic efficacy and application are below expectations. In this study, a glutathione (GSH)-responsive nano-photosensitizer, based on the chlorquinaldol (CQD)-loaded iron-containing nanorod composed of meso-tetra (4-carboxyphenyl) porphyrin (TCPP), was prepared to serve as the laser-ignited ferroptosis sensitizer to improve the tumoricidal effect of PDT. In the tumor microenvironment (TME) with elevated GSH levels, therapeutic cargos and ferrous ions are released and are accompanied by the degradation of the nano-photosensitizer and GSH exhaustion. This not only increases liable iron pool (LIP) accumulation by the released ferrous ions but also decreases glutathione peroxidase 4 (GPX4) activity by GSH exhaustion. Simultaneously, GSH exhaustion disrupts intracellular redox homeostasis, heightening NIR light irradiation-triggered photosensitive oxidative stress. Moreover, the released CQD elevates the level of intracellular reactive oxygen species (ROS), enabling the nanorods to gain an oxygen radical generation ability and enhancing the photosensitive oxidative therapeutic efficacy. Strikingly, CQD exacerbates the downregulation of GPX4 expression to promote the accumulation of lipid peroxides. Therefore, we herald a new paradigm for synergistically potentiating PDT based on the “all-in-one” nano-photosensitizer through the multi-pronged upregulation of ferroptosis sensitivity.

## 1. Introduction

Colorectal cancer (CRC) represents a significant global health challenge, ranking third in cancer incidence and second in mortality worldwide [1,2,3]. Despite surgical resection being the standard treatment, it often compromises the patient quality of life, necessitating innovative therapeutic approaches [4]. Photodynamic therapy (PDT), as an adjuvant therapy, has entered clinical trials for CRC treatment [5] and has emerged as a promising alternative, leveraging photosensitizers to generate reactive oxygen species (ROS) through site-specific laser irradiation, inducing targeted cytotoxicity by oxidizing biological macromolecules. The therapeutic potential of PDT depends on its unique characteristics: non-invasiveness, spatial-temporal selectivity, and minimal side effects [6,7,8]. Thus, PDT offers complementary and synergistic treatment possibilities. However, its clinical efficacy is substantially constrained by critical limitations, including profound hypoxia in the tumor microenvironment (TME), limited light tissue penetration, and the transient nature of ROS generation [9,10]. Recent advances in nanomedicine have catalyzed innovative approaches and focused on the development of multifunctional nanoplatforms and sensitization strategies to achieve the improved antitumor effects of PDT, particularly the intersection of PDT with ferroptosis [11,12,13,14]. It is noted that PDT was able to trigger ferroptosis-like cell death pathways through membrane lipid peroxidation [15]. This implies that upregulating the sensitivity of ferroptosis is expected to promote a PDT curative effect.

Ferroptosis is an iron-dependent form of cell death marked by excessive lipid peroxidation and membrane impairment [16]. Mechanistically, ferroptosis involves intricate iron-mediated processes. The labile iron pool (LIP) in the cellular microenvironment plays a crucial role, where excessive free ferrous ions exacerbate the formation of ROS through Fenton reactions [17,18]. Thus, it is rational to speculate that the addition of exogenous ferrous ions disrupts iron homeostasis and creates an oxidative cascade that ultimately induces ferroptosis. Glutathione peroxidase 4 (GPX4) is pivotal in maintaining membrane lipid integrity by catalyzing the reduction of lipid hydroperoxides (LOOH) to lipid alcohols (LOH) through a glutathione (GSH)-dependent pathway [18,19,20]. The overexpression or knockdown modulation of cellular sensitivity to ferroptosis suggests GPX4 is a critical resistance factor to ferroptosis [21]. Notably, functional studies in melanoma and non-small cell lung cancer have demonstrated that GPX4 inactivation can induce persistent tumor ferroptosis in vitro, suggesting its potential as a critical therapeutic target [22]. Emerging evidence demonstrates significant GPX4 overexpression in various malignant tissues, including colon and rectal adenocarcinomas and prostate cancer, contrasting with normal tissue levels [23]. Thus, dysregulated GPX4 contributes to ferroptosis with significant implications for potentially overcoming tumor drug resistance. By compromising the enzyme’s ability to scavenge lipid peroxides, researchers can strategically trigger ferroptosis. Consequently, multiple ferroptosis inducers targeting GPX4 have been developed, including RSL3 [24], ML162, C18 [25], and FIN56 [26]. Despite these promising advances, significant challenges persist. Typically, the poor pharmacokinetic properties and potential off-target effects of current GPX4 inhibitors substantially limit their clinical translation [27,28]. Therefore, the modification of existing inhibitors and exploration of the biological effects of existing drugs on GPX4 are urgently required for overcoming these obstacles.

Chlorquinaldol (CQD), a derivative of 8-hydroxyquinoline, has historically been recognized as a topical antimicrobial agent for dermatological use and is frequently employed in conjunction with probenecid for the treatment of vaginitis [29,30]. Despite its extensive clinical application in skin therapeutics, its potential anticancer properties remained largely unexplored until recent investigations. CQD combats colorectal cancer by disrupting β-catenin and T-cell factor 4 complexes. It also curtails CRC cell proliferation, migration, invasion, and stemness [31]. Additionally, the iron complex of CQD can induce antitumor effects by promoting apoptosis via cell cycle arrest and DNA damage [32]. As a lipophilic Fe(III)-chelator, CQD enhances cellular uptake, disrupts the mitochondrial membrane potential, and promotes the production of ROS in tumor cells, thus enhancing anticancer activity by utilizing ferroptosis [33]. There are limited studies on the relationship between CQD and ferroptosis, which deserves further exploration.

**Scheme 1:**
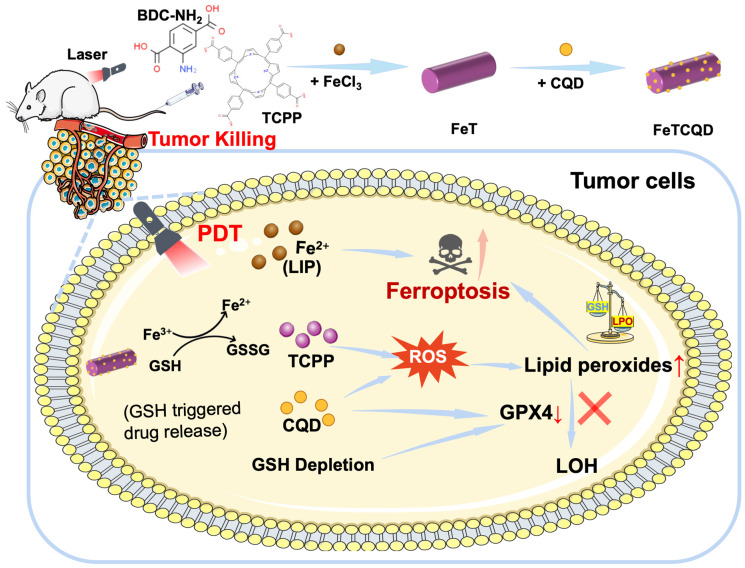
Schematic representation of the nanorod FeTCQD synthesis and GSH-triggered drug release for cancer treatment.

Herein, we developed CQD-loaded iron porphyrin nanorods (FeTCQD) with dual synergistic effects against cancer cells. Utilizing a hydrothermal synthesis approach, FeT nanorods were constructed by coordinating iron chloride, 2-amino-terephthalic acid (BDC-NH_2_), and meso-tetra (4-carboxyphenyl) porphyrin (TCPP), which were subsequently loaded with CQD. Upon cellular internalization, FeTCQD underwent degradation in the tumor microenvironment, characterized by increased GSH levels, initiating a cascade of therapeutic interventions. The released Fe^3+^ ions were rapidly reduced to ferrous ions leading to LIP accumulation. Accompanying GSH exhaustion not only disrupted intracellular redox homeostasis but also led to a decrease in GPX4 synthesis. These together with the reduced level of GPX4 protein by the released CQD further resulted in the generation of toxic PL-PUFA-OOH, finally accelerating the occurrence of ferroptosis. On the other hand, the PDT process mediated by TCPP could generate singlet oxygen (^1^O_2_) when exposed to a 660 nm laser, which can directly kill tumors. Meanwhile, PDT elicited a catastrophic lipid ROS storm that led to a large accumulation of toxic PL-PUFA-OOH, further amplifying ferroptosis. These synergistic effects dramatically improve the tumoricidal efficiency of PDT. Both in vitro and in vivo studies demonstrated remarkable antitumor efficacy of the synthesized FeTCQD (Scheme 1). Therefore, we present the GSH-responsive nanorods, FeTCQD, that can enhance ferroptosis and PDT by effectively increasing LIP and impairing GPX4 activity, offering an innovative strategy to improve PDT effectiveness and antitumor treatment.

## 2. Materials and Methods

### 2.1. Materials

TCPP and BDC-NH_2_ were obtained from Macklin, while the malondialdehyde (MDA) assay kit was sourced from Shanghai Beyotime Biotechnology Co., Ltd. (Shanghai, China). Ferrostatin-1 (Fer-1) and CQD were procured from TargetMol Biotech Co., Ltd., (Shanghai, China) and the GSH assay kit was bought from Solarbio Biotechnology Co., Ltd. (Beijing, China). Additionally, BALB/c nude mice were acquired from SPF Vital River Laboratories Technology Co., Ltd. (Beijing, China).

### 2.2. Synthesis of FeTCQD

TCPP (0.05 g), BDC-NH_2_ (0.11 g), and FeCl_3_·6H_2_O (1.35 g) were dissolved in 75 mL DMF and transferred into an autoclave for 6 h of reaction at 90 °C. After centrifugation, the obtained sediment (FeT) was washed three times to remove the excess reactants. To prepare the FeTCQD, 1 mg FeT was mixed with 100 μg CQD. After stirring for 24 h, FeTCQD was obtained after washing.

### 2.3. Characterizations

The morphology of FeTCQD was conducted using transmission electron microscope (TEM) (Tecnai F20 TEM instrument, Hillsboro, OR, USA). Infrared spectra for both FeTCQD and TCPP were recorded by the Fourier-transform infrared spectroscopy (FT-IR) using a Nicolet iS10 from Thermo Fisher Scientific, Waltham, MA, USA. Dynamic light scattering (DLS) size distribution was measured on the Zetasizer Nanoseries (Malvern, Malvern City, UK).

### 2.4. GSH-Mediated Detection of FeTCQD in Solutions

The GSH solution (10 mM) was incubated with FeTCQD at different concentrations dispersed in PBS. The residual GSH was measured by a GSH detection kit after centrifugation. In addition, the amount of GSH consumed by FeTCQD at different time points (1, 3, 6, 12, and 24 h) was also determined, following a similar procedure as above.

### 2.5. Release of Iron Ions from Nanorods FeTCQD

The reaction of FeTCQD with GSH was carried out at different times or in different concentrations of the GSH solution. Once the reaction was halted, the supernatant was collected and 0.1% of phenanthroline was added. Then, the OD value (512 nm) was measured, and the concentration of the ferric iron ions was calculated.

### 2.6. Cell Culture and Cellular Uptake

HCT116 cells were kindly gifted by Dr. Desheng Lv from Shenzhen University Medical School. HCT116 cells were cultured in a complete RPMI 1640 cell culture medium with 10% fetal bovine serum in a 37 °C incubator with 5% CO_2_ atmosphere. The uptake and degradation of FeTCQD in cells were investigated by cellular uptake experiments. The process can be observed by detecting the fluorescence signal of TCPP due to the fluorescence property of TCPP itself. The fluorescent signal of TCPP was detected by adding FeTCQD (100 μg mL^−1^) into the confocal dish inoculated with HCT116 cells, and the fluorescence uptake of the cells was observed after incubation for 0, 1, and 3 h.

### 2.7. Cytotoxicity Evaluation

Cell viability was assessed by CCK-8 assay. HCT116 cells were incubated for 24 h in 96-well plates and processed with FeTCQD at varying concentrations (12.5–100 μg mL^−1^). Then, the cells were subjected to a 5 min exposure to a 660 nm laser or left untreated after 12 h of incubation. Following 24 h of incubation, CCK-8 agent was added to the medium and the absorbance at 450 nm for each well was determined.

### 2.8. Intracellular Iron Ion Release Measurement

HCT116 cells were inoculated into confocal dishes, and free CQD, FeT, or FeTCQD (TCPP concentration: 50 μg mL^−1^) was added into separate confocal dishes and incubated for 6 h (the FeTCQD+DFO group was pretreated with 200 μM deferoxamine (DFO) for 2 h in advance). The cells were washed and incubated with FerroOrange working solution for 30 min, and then CLSM was used to observe the fluorescence.

### 2.9. GPX4 Analysis

The cells were incubated with CQD, FeT, and FeTCQD separately. After 24 h of irradiation, proteins were extracted and subjected to standard electrophoresis in 10% SDS-PAGE gels. Briefly, the samples were transferred to polyvinylidene fluoride membranes after electrophoresis. The membranes were then blocked with 5% non-fat milk and incubated overnight with anti-GPX4 primary antibodies at 4 °C. Afterward, horseradish peroxidase-conjugated secondary antibodies were employed to perform chemiluminescence imaging. Beta-actin was used as a loading control to normalize the target protein.

### 2.10. Intracellular MDA Measurement

HCT116 cells were inoculated into culture dishes. After the number of cells in each group reached 5 × 10^6^, each experimental treatment group with a TCPP concentration of 50 μg mL^−1^ was added into the dishes and incubated with the cells for 12 h. The light group received laser exposure for 5 min and then continued to be incubated overnight. The MDA content of the cells was measured and calculated according to the requirements of the MDA assay kit.

### 2.11. Tumor Models

Five-week-old BALB/c nude mice were injected subcutaneously with 5 × 10^6^ HCT116 cells to implant the tumors. The tumor size was measured with a vernier caliper, with the tumor volume calculated using the formula V = L/2 × W^2^ (L: length, W: width). When the tumor volume reached about 100 mm^3^, the mice were weighed and categorized into four groups (n = 5). They received intravenous injections with PBS, CQD, FeT, and FeTCQD (at an identical TCPP dose of 5 mg/kg) every 3 days. At 12 h post-injection, the tumor site was irradiated with a laser for 5 min (660 nm, 200 mW cm^−2^) in the FeT and FeTCQD group, and the weight and tumor size of the mice were measured every 2 days. After 14 days, all the mice were sacrificed, and the tumor samples were stripped and weighed. The obtained tumor tissues were fixed with 10% formalin and embedded with paraffin. After immunohistochemical staining with the primary antibodies against GPX4, Ki67, 4-HNE, and H&E staining, tissue sections were observed under the Olympus IX73 microscope.

## 3. Results and Discussion

### 3.1. Synthesis and Characterization of FeTCQD

**Figure 1:**
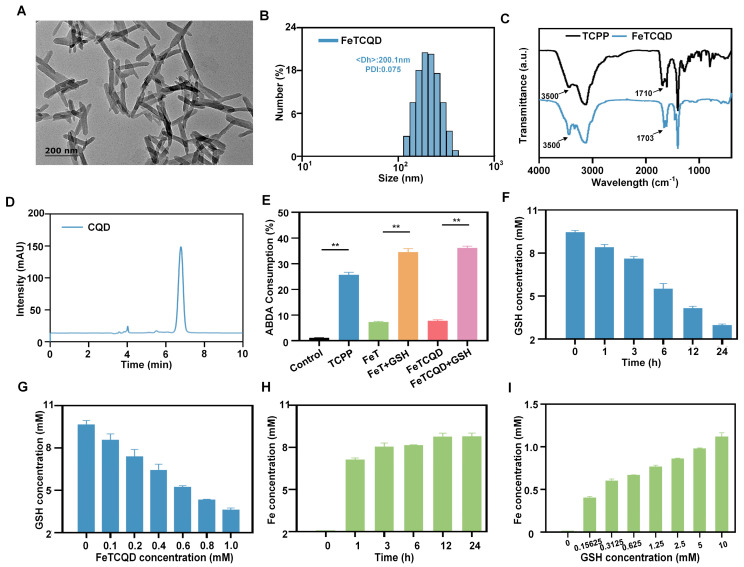
(**A**) TEM image of FeTCQD nanorods. (**B**) Hydrodynamic size distributions and PDI analysis of FeTCQD measured by DLS. (**C**) FT-IR spectra of TCPP and FeTCQD. (**D**) HPLC analysis of FeT loaded CQD. (**E**) Singlet oxygen detection of FeT and FeTCQD with and without laser irradiation by ABDA probe (660 nm, 100 mW cm^−2^, 5 min), n = 5. (**F**) Detection of GSH concentration in aqueous solutions at different times with 1 mg mL^−1^ FeTCQD treatment, n = 5. (**G**) GSH levels treated with different concentrations of FeTCQD in aqueous solutions for 24 h, n = 5. (**H**) Concentration of iron ions in solutions at different incubation times, n = 5. (**I**) Iron ion concentrations in solutions at different GSH concentrations, n = 5. ** *p* < 0.01.

Given nitrogen’s exceptional ability to coordinate with Fe [34], TCPP and BDC-NH_2_ were selected as dual ligands to interact with the iron ions. Herein, the porous FeT was successfully synthesized through a straightforward one-step hydrothermal method utilizing Fe(III) salt, TCPP, and BDC-NH_2_ at 90 °C for 6 h, and its unique porous structure allowed for the possibility of carrying CQD by physical adsorption. The FeTCQD images from TEM showed the monodispersed uniform shape of rod morphology with a diameter of ~180 nm (Figure 1A). DLS revealed that the polydispersity index (PDI) of FeTCQD was 0.074, indicating high uniformity with a mean diameter of 200.1 nm (Figure 1B), which was consistent with TEM images.

FT-IR provided the critical evidence of TCPP incorporation. The characteristic C=O stretching vibration observed at 1703 cm^−1^ (Figure 1C) substantiated the metal ion coordination, a key structural feature of the nanorods. To assess whether CQD was successfully loaded, high-performance liquid chromatography (HPLC) was employed to analyze the CQD content in the supernatant, enabling precise determination of the CQD content according to the total amount of CQD. (Figure 1D and Figure S1). Extensive optimization efforts focused on maximizing encapsulation efficiency revealed the optimal conditions at a 10:1 mass ratio of FeT to CQD. Under these parameters, the nanorods achieved a remarkable 69% encapsulation efficiency, with a loading content of 6.5% (Table S1). These comprehensive characterization results collectively validate the successful synthesis of the FeTCQD nanorods.

Photosensitization is responsible for the efficacy of PDT. To characterize the photosensitive effect, the ABDA probe was leveraged as an indicator for ^1^O_2_ determination. As displayed in Figure 1E, the ^1^O_2_ levels were notably increased in both the FeT and FeTCQD groups when incubated with GSH compared to the other groups. Conversely, the levels of ^1^O_2_ of FeT or FeTCQD alone were well below that of the TCPP group, which also implied that the disintegration of FeTCQD was reliant on the presence of GSH.

To evaluate the TME responsiveness of FeTCQD, we conducted a comprehensive series of experiments investigating its interaction with GSH. Utilizing a GSH detection kit, we quantified the residual GSH concentrations in the supernatant under varying nanorod concentrations and incubation times. As illustrated in Figure 1F,G, a systematic dose- and time-dependent depletion of GSH was observed. The progressive reduction in GSH concentration demonstrated the remarkable capability of FeTCQD to effectively disrupt cellular redox homeostasis. This GSH depletion mechanism is critical for initiating the proposed therapeutic strategy, highlighting the nanorods’ potential to compromise the antioxidant defenses of cancer cells. Complementary to GSH analysis, we quantified iron ion release using phenanthroline spectrophotometry under identical experimental conditions. This additional characterization provided further insight into the nanorods’ dynamic interactions within the cellular microenvironment. As illustrated in Figure 1H,I, the iron ion levels in the supernatant rose with the augmentation of GSH concentration and incubation time. In summary, the synthesized FeTCQD was stable in a GSH-free environment, while GSH played a crucial role in triggering the release of CQD and TCPP, which were conducive to its subsequent in vitro and in vivo application.

### 3.2. Cellular Uptake and Antitumor Effects of FeTCQD In Vitro

**Figure 2:**
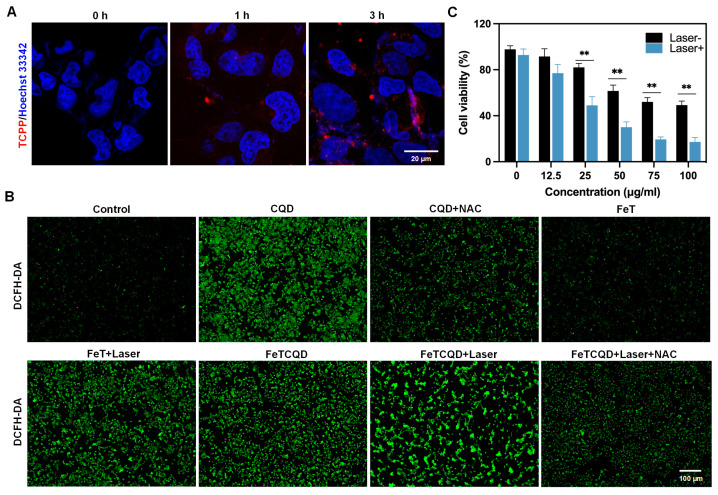
(**A**) Images of cellular uptake of FeTCQD at different incubation times. (**B**) Images of HCT116 cells post various treatments for ROS detection. (**C**) Cell viability across varying FeTCQD concentrations with and without laser exposure (660 nm, 50 mW cm^−2^, 5 min), n = 5. ** *p* < 0.01.

The uptake of the prepared nanorods was monitored by tracking the red fluorescence emitted by TCPP released from FeTCQD, and the nucleus was stained blue with Hoechst 33342 in HCT116 cells. Confocal microscopy results demonstrated that the fluorescence intensity of TCPP rose progressively over time, suggesting effective cellular uptake and the accumulation of FeTCQD (Figure 2A).

To validate the restoration of intracellular TCPP photosensitization, the capacity of FeTCQD to generate ROS in cells under different treatments was investigated with 2′, 7′ -dichlorofluorescein diacetate (DCFH-DA), and the intracellular ROS levels were evaluated by confocal fluorescence imaging. It could be observed that treatment with CQD, FeT, and FeTCQD after laser irradiation all had strong green fluorescence, while the addition of the antioxidant NAC reduced the corresponding fluorescence intensity (Figure 2B). In all groups, the fluorescence intensity of FeTCQD under laser irradiation was significantly higher than that of other groups, which was attributed to the synergistic effect of PDT and CQD. These results indicated that FeTCQD with laser irradiation can produce more intracellular ROS than other groups.

Subsequently, CCK-8 assays were performed in HCT116 cells to investigate the cytotoxicity of FeTCQD. The results indicated that the cell viability showed a dose-dependent decline in FeTCQD with or without laser irradiation. Importantly, HCT116 cells treated with 100 μg mL^−1^ FeTCQD under the laser-off condition had a survival rate of approximately 50% due to the anti-cancer activity of CQD itself. In contrast with the laser-off group, FeTCQD with laser-on presented much higher cytotoxicity owing to the synergistic effect of PDT and CQD (Figure 2C). According to the above results, we concluded that the FeTCQD nanorods can be phagocytosed by tumor cells to produce ROS and show a strong capability to subvert tumor cells after irradiation.

### 3.3. Mechanism of FeTCQD-Induced Cell Death In Vitro

**Figure 3:**
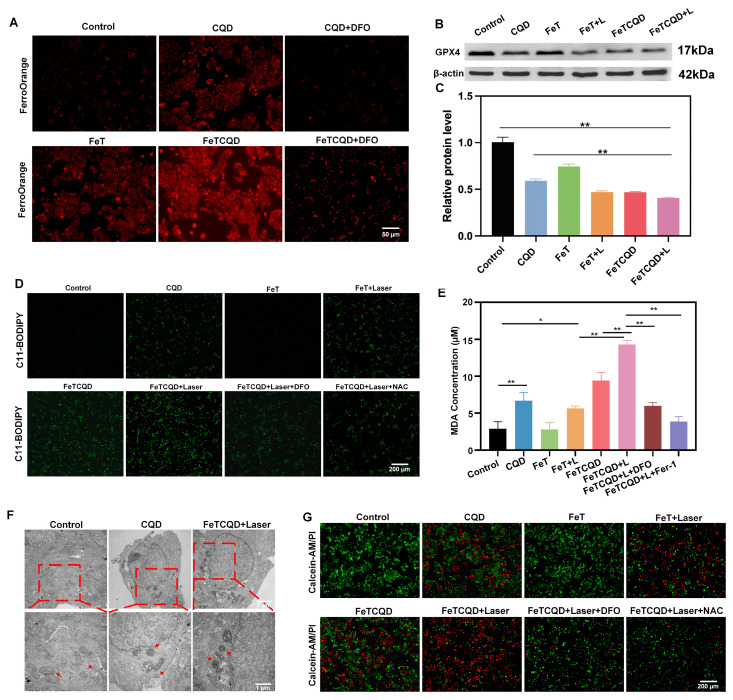
(**A**) Images of Fe^2+^ release in cells incubated with FeTCQD. (**B**) Western blot analysis of GPX4 expression in cells with different treatments and (**C**) corresponding quantification, n = 3. (**D**) Images for intracellular lipid peroxidation with different treatments by C11-BODIPY. (**E**) Intracellular MDA levels with different treatments, n = 5. (**F**) Bio-TEM images of HCT116 cells subjected to varying treatments, and the arrows indicate mitochondria. (**G**) Live/dead staining assay cells of HCT116 cells post-incubation with CQD, FeT, and FeTCQD without or with laser irradiation (green: live cells; red: dead cells). * *p* < 0.05, ** *p* < 0.01.

FeTCQD nanorods were taken up by tumor cells and disintegrated in the TME, releasing various components including iron ions. The release of ferrous ions from FeTCQD nanorods was observed by CLSM. The red fluorescence of FeTCQD was the strongest in all the treatment groups, and the addition of the iron chelator and ferroptosis inhibitor DFO could reverse this phenomenon (Figure 3A), verifying that ferrous ions could be produced after the uptake of FeTCQD by cells. Meanwhile, the results showed that treatment with CQD alone also increased the intracellular iron ion levels, except for promoting the production of ROS. Therefore, we further investigated the effects of CQD and FeTCQD on ferroptosis.

GSH is an important cofactor in the detoxification of lipid peroxidation by GPX4. We hypothesized that its depletion could indirectly induce the inhibition of GPX4 and lead to the accumulation of lipid peroxidation. To demonstrate this, GPX4 expression was detected by Western blot analysis. As expected, both CQD and FeTCQD alone could decrease the levels of GPX4, and the GPX4 expression level was lowest in FeTCQD-treated cells (Figure 3B,C), mainly depending on the increased GSH consumption and the released CQD. This result suggests that the synergistic effect of GSH depletion and CQD may further impair cellular antioxidant defenses.

Lipid peroxidation serves as a key metric of ferroptosis [19]. Here, a specific fluorescent probe C11-BODIPY was leveraged to detect the accumulation of lipid peroxidation under different treatments. As presented in Figure 3D, the bright green fluorescence indicated the presence of lipid peroxidation following various cell treatments. A notable rise in lipid peroxidation production was observed in cells treated with either FeT or FeTCQD after laser exposure compared to the non-laser exposure groups. Notably, the FeTCQD+Laser group produced higher levels of lipid peroxidation relative to the FeT+Laser group, probably resulting from the collaborative function of CQD itself. The addition of the antioxidant NAC and the iron-chelating agent DFO both weakened this phenomenon, which was in accordance with the above speculation. In addition, we measured the levels of the lipid peroxidation product MDA [35]. The results showed that treatment with CQD alone could significantly enhance the level of MDA, suggesting that CQD has the ability to induce ferroptosis. It was also observed that the MDA level in the FeTCQD+Laser group was highest when contrasted with the other groups, and the ferroptosis inhibitors DFO and Fer-1 both reversed the increased effects induced by FeTCQD after irradiation (Figure 3E). Together, we concluded that the nanorods significantly enhance lipid peroxidation accumulation in tumor cells, consequently resulting in a notable increase in MDA levels.

Furthermore, mitochondria play a crucial role in the occurrence of ferroptosis and are morphologically unique. Thus, cell morphology was examined by biological electron microscopy to further confirm the occurrence of ferroptosis. As illustrated in Figure 3F, the cell morphology of the control group remained normal, whereas the cells in the CQD and FeTCQD+Laser groups exhibited the typical features of ferroptosis, including mitochondrial atrophy and the disappearance or reduction in cristae.

For a more straightforward illustration, cell viability in different treatment groups was analyzed using the Calcein-AM/PI assay kit. As shown in Figure 3G, the red fluorescence denoting cell death was observed in CQD, FeT, and FeTCQD groups following laser exposure. Compared with the other groups, FeTCQD+Laser treatment significantly reduced the cell viability. At the same time, the corresponding fluorescence was significantly inhibited after cotreatment with DFO or NAC, which proved that ferroptosis probably played an important role in FeTCQD-induced cell death. Together, these results demonstrated that FeTCQD efficiently induces cell ferroptosis through multi-pronged synergistic effects including Fe^2+^ accumulation, GPX4 downregulation, and lipid peroxidation.

### 3.4. In Vivo Antitumor Effect of FeTCQD

**Figure 4:**
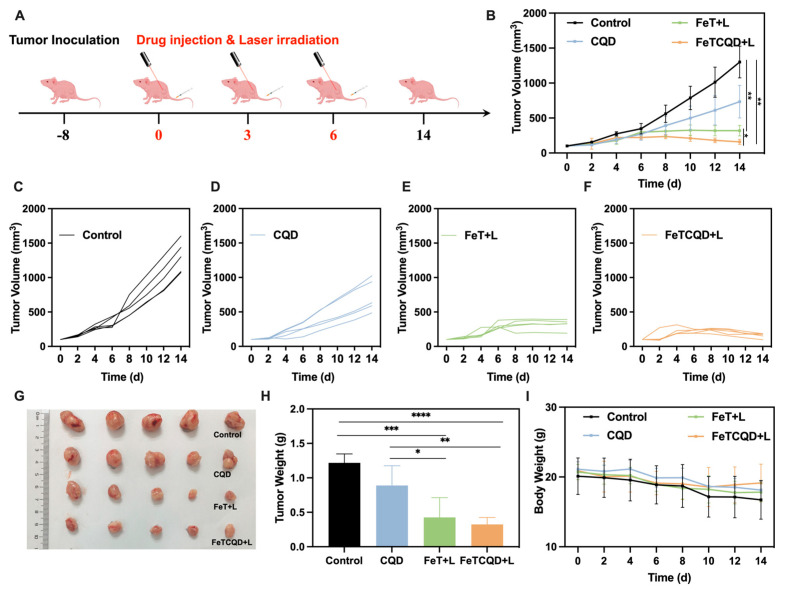
(**A**) Scheme illustrating the establishment and treatment of the tumor-bearing mice model. (**B**) Tumor volume curves of the different treatments, n = 5. (**C**–**F**) Growth curves for individual tumors in four groups, n = 5. (**G**) Photographs of the dissected tumor tissues on the 14th day. (**H**) Tumor weight on the 14th day post-treatments, n = 5. (**I**) Body weight changes of the mice over a 14-day treatment period, n = 5. * *p* < 0.05, ** *p* < 0.01, *** *p* < 0.001, **** *p*< 0.0001.

To further evaluate the antitumor efficacy of FeTCQD in vivo, HCT116-tumor-bearing mice were randomly assigned into four groups: Control, CQD, FeT, and FeTCQD. The mice received intravenous injections at 0, 3, and 6 days with three doses administered in total. Following each injection, laser therapy was administered 12 h later (Figure 4A). Tumor volumes were measured every two days using a caliper. Compared with other groups, the FeTCQD+L group had significantly inhibited tumor growth (Figure 4B–F), indicating that FeTCQD exhibits a pronounced antitumor effect after laser irradiation. Consistent conclusions were also derived from the tumor images and tumor weights (Figure 4G,H). The body weight changes of the mice were recorded during the treatment period (Figure 4I), and the FeTCQD+L group did not exhibit a marked weight change relative to the control group, indicating negligible toxicity of the FeTCQD.

**Figure 5:**
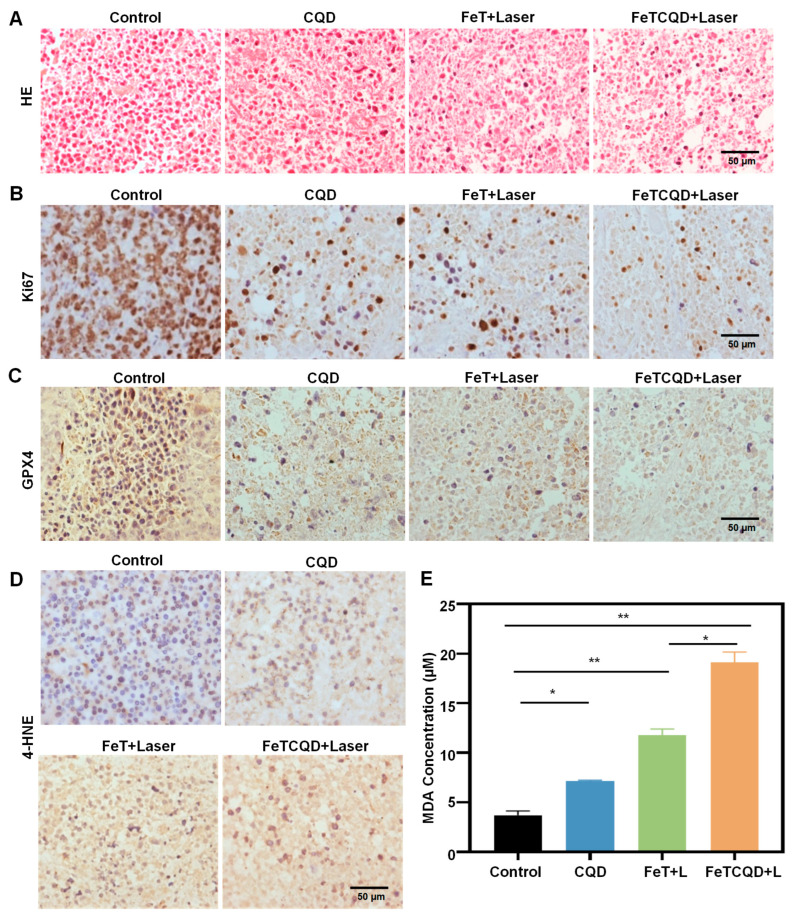
(**A**) H&E and (**B**) Ki67 staining of tumor tissues after different treatments. (**C**) GPX4 and (**D**) 4-HNE staining of tumor tissues after different treatments. (**E**) MDA content of tumor tissues after different treatments. * *p* < 0.05, ** *p* < 0.01.

In addition, the tumor sections were subjected to pathological analysis. The H&E staining results for the control group showed a dense arrangement with high vitality, while nuclear fragmentation and reduction were observed in the FeTCQD+L group (Figure 5A). Immunohistochemical staining with Ki67 antibodies further showed an increase in dead cancer cells and a significant inhibition of cell proliferation following FeTCQD+L treatment (Figure 5B), showing the excellent antitumor effect of FeTCQD compared to FeT under laser irradiation.

The related indicators of ferroptosis including GPX4, MDA, and 4-HNE were also detected in tumor tissues. Compared with the other groups, the FeTCQD+L group notably reduced the expression of GPX4 and elevated the levels of 4-HNE and MDA, another lipid peroxidation product of ferroptosis (Figure 5C–E). Notably, compared with the control group, treatment with CQD also suppressed GPX4 expression which was accompanied by increased levels in MDA and 4-HNE. These results demonstrate that the designed FeTCQD can improve the tumoricidal efficacy of PDT through the enhanced inhibition of GPX4 and lipid peroxidation accumulation.

## 4. Conclusions

In this study, a GSH-responsive drug carrier loaded with CQD was rationally fabricated, which achieved an orchestrated synergy of ferroptosis and PDT for enhancing the antitumor therapeutic effect. The FeTCQD nanorods enhanced the effective concentration of TCPP at the tumor locations and raised the intracellular LIP concentration based on the properties of the GSH-responsive release. Simultaneously, GSH exhaustion disrupted intracellular redox homeostasis, heightening the NIR light irradiation-triggered photosensitive oxidative stress. Moreover, the released CQD demonstrated the ability to significantly upregulate ROS generation and downregulate GPX4 expression, resulting in accelerating the occurrence of ferroptosis. Almost all GPX4 inhibitors are alkylating agents, which have low selectivity and poor pharmacokinetics, and will also cause drug resistance. CQD is an old anti-infection drug with safety, which could promote the levels of ROS and ferrous ions in tumor cells, while inhibiting GPX4 and increasing oxidative stress. PDT can only act on superficial tumor sites, and based on this strategy, it is possible to extend the treatment of tumors. Taken together, the FeTCQD nanorods are promising to serve as an excellent ferroptosis nano-inducer for improving the efficacy of PDT and upregulating ferroptosis sensitivity by a multi-pronged synergistic mechanism.

## Data Availability

Data will be made available on request.

## Supplementary Materials

The following supporting information can be downloaded at: https://www.mdpi.com/article/10.3390/antiox14040407/s1, Figure S1: Standard absorption curve of CQD solution at 250 nm by HPLC; Table S1: The encapsulation efficiency of FeT-loaded CQD.

## Abbreviations

PDT	Photodynamic therapy
CQD	Chlorquinaldol
GPX4	Glutathione peroxidase 4
LIP	Liable iron pool
TCPP	Meso-tetra (4-carboxyphenyl) porphyrin
