# Supplementary material for: GSH-Responsive Nano-Photosensitizer for Potentiating Photodynamic Therapy Through Multi-Pronged Synergistic Upregulation of Ferroptosis Sensitivity
Source: Antioxidants (Basel). 2025 Mar 28;14(4):407. doi: 10.3390/antiox14040407 (PMC12024321; doi:10.3390/antiox14040407)
Supplement: Supplementary file 1 [file antioxidants-14-00407-s001.zip › antioxidants-3518780-supplementary.pdf]

Supplementary Materials

GSH-Responsive Nano-Photosensitizer for Potentiating Photodynamic Therapy Through Multi-Pronged Synergistic Upregulation of Ferroptosis Sensitivity

Yunong Ma [1,†], Kexin Xu [1,†], Jing Feng [1], Xi Zhao [1], Peilin Tian [1], Jiayang Luo [1], Luyao Xu [1], Jiaxing Song [2,*] and Cuixia Lu [1,*]

1 Guangxi Key Laboratory of Special Biomedicine; School of Medicine, Guangxi University, Nanning 530004, China; mynnong@163.com (Y.M.); echooo@st.gxu.edu.cn (K.X.); fengj@st.gxu.edu.cn (J.F.); zxnl2022@163.com (X.Z.); tianpl1216@st.gxu.edu.cn (P.T.); jiayungl@st.gxu.edu.cn (J.L.); luluna@st.gxu.edu.cn (L.X.)

2 Cell and Immunology Laboratory, Medical Research Centre, School of Life Sciences and Medical Engineering, Guangxi Medical University, Nanning 530021, China

* Correspondence: songjiaxing@gxmu.edu.cn (J.S.); lucuixia@gxu.edu.cn (C.L.)

† These authors contributed equally to this work.

Materials and Methods

ROS Production by Nanorods FeTCQD in Aqueous Solutions

TCPP, FeT and FeTCQD were mixed with GSH solution (10 mM) in equal volume (Control group was added with the same volume of double distilled water). The reaction was carried out on a shaking table for 24 h. The supernatant was discarded and DMSO containing 9,10-anthracenediyl-bis (methylene) dicarboxylic acid (ABDA) was added, and the laser group was irradiated for 5 min (laser condition: 100 mW cm^{-2}, 660 nm). Absorbance at 378 nm was quantified and ABDA reduction (%) was then determined per group.

In Vitro ROS Generation

Cells were incubated with fresh media containing CQD, FeT, and FeTCQD (equivalent to 50 μg mL^{-1} TCPP) respectively for 12 h. After washing with PBS, each treatment group was incubated with 2',7'-dichlorofluorescein diacetate (DCFH-DA) for 30 min, and the laser group was irradiated with laser for 5 min (50 mW cm^{-2},660 nm). Finally, the fluorescence intensity of DCF was immediately observed by confocal laser scanning microscope (CLSM).

Intracellular Lipid Peroxide Measurement

The C11-BODIPY 581/591 probe was utilized to assess the intracellular accumulation of lipid peroxidation. HCT116 cells were incubated with CQD, FeT and FeTCQD and subsequently irradiated with 660 nm laser. 4 h post-irradiation, the cells were incubated with the probe for 1 h and then imaged using CLSM.

Live/Dead Assay

The therapeutic efficacy was evaluated using Calcein-AM/PI staining. HCT116 cells were incubated with free CQD, FeT, or FeTCQD (TCPP concentration: 50 μg mL^{-1}) in 24-well plates for 12 h, then washed twice with PBS and replaced with fresh medium, either irradiated with 660 nm laser (50 mW cm^{-2}) for 5 min or left untreated.

To investigate the effect of ferroptosis inhibition on the cytotoxicity of CQD, FeT, or FeTCQD under laser irradiation, different ferroptosis inhibitors, including DFO (200 μM) and N-acetylcysteine (NAC), were added. After a 24 h incubation period, living and dead cells were labeled with Calcein-AM (2 μM) and PI (4 μM) for 15 min, respectively. Finally, the green and red fluorescence from Calcein-AM and PI in HCT116 cells was observed with EVOS cell imaging system.

Supporting Figures

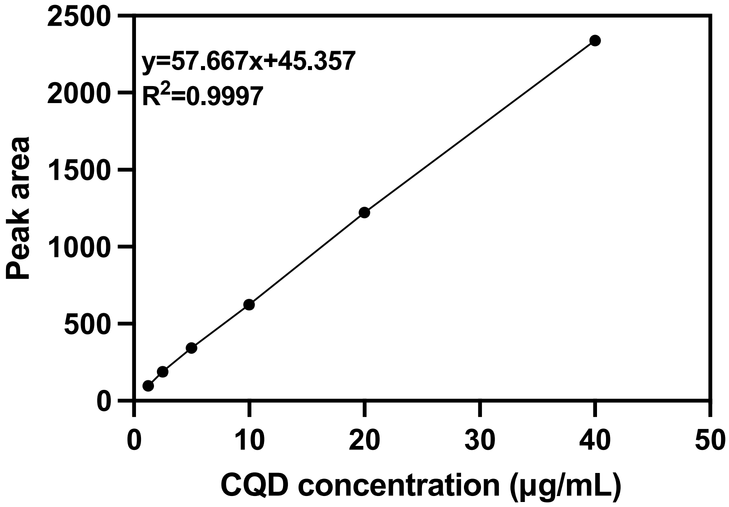

Figure S1. Standard absorption curve of CQD solution at 250 nm by HPLC.

Table S1. The encapsulation efficiency of FeT loaded CQD.

Number	M (FeT:CQD)	Encapsulation efficiency	Loading efficiency
1	1:1	21.9 %	18.0 %
2	1:0.2	61.5 %	10.9 %
3	1:0.1	69.0 %	6.5 %
4	1:0.05	65.2 %	3.2 %
5	1:0.025	42.4 %	1.1 %
